# Fertility-sparing management of perivascular epithelioid cell tumors: A case report and literature review

**DOI:** 10.1016/j.crwh.2025.e00766

**Published:** 2025-11-14

**Authors:** Laura Jore, Nneoma Edokobi, Ishani Deliwala, Marykate Potter, Meg Johnson, LucyBeth Nieves

**Affiliations:** Novant Health New Hanover Regional Medical Center, Wilmington, NC, USA

**Keywords:** Gynecologic perivascular epithelioid cell tumor, Fertility preservation, Oncofertility

## Abstract

Perivascular epithelioid cell tumors (PEComas) are rare mesenchymal neoplasms. Approximately 25 % occur in the female reproductive tract. The treatment strategy suggested in the literature is largely limited to hysterectomy, with little consideration of fertility-sparing approaches in reproductive-aged patients. This report concerns the use of a fertility-sparing approach in the management of a nulligravid woman who presented with abnormal uterine bleeding. She was diagnosed with a cervical perivascular epithelioid cell tumor based on biopsy and immunohistochemical analysis. Imaging confirmed a localized cervical mass and an endometrial lesion. The patient underwent hysteroscopic removal of the endometrial mass. Loop electrosurgical excision was utilized for the cervical tumor. Pathology demonstrated a benign cervical perivascular epithelioid cell tumor and an endometrial polyp. The patient remained disease-free with negative pelvic magnetic resonance imaging at three, six, and twelve months. This case highlights a novel use of loop electrosurgical excision to preserve fertility in those with gynecologic tumors, although ongoing surveillance is essential to ensure long-term safety.

## Highlights

• Gynecologic perivascular epithelioid cell tumor management strategies do not emphasize fertility sparing techniques.
• Fertility sparing management of benign cervical perivascular epithelioid cell tumors via LEEP is feasible.
• Novel use of LEEP in cervical perivascular epithelioid cell tumor contributes to oncofertility preservation literature.
• Close surveillance is critical after fertility sparing management of gynecologic perivascular epithelioid cell tumors.

## 1 Introduction

Perivascular epithelioid cell tumors (PEComas) are a rare group of mesenchymal neoplasms first described by Zamboni et al. [1] in 1996. They develop in various organs, including the lung, kidney, liver, small and large bowel, and reproductive tract [2]. Approximately 25 % occur in the female reproductive tract. Among gynecologic PEComas, the majority originate in the uterine corpus (20 %), while cervical, adnexal, and pelvic ligament involvement remains rare [3]. Levin's [4] literature review described fifty-eight case reports of gynecologic PEComas in the English literature, encompassing eighty-four patients. Uterine involvement was found in 81 % of cases, cervical involvement in 9 %, ovarian involvement in 6 %, and vaginal involvement in 4 % [4].

Histopathologic classification categorizes PEComas as benign, uncertain malignant potential, or malignant. This classification is particularly relevant in the context of fertility-sparing approaches, which have been documented only in select cases of benign and uncertain malignant potential PEComas, and in malignant cases where surgical resection was not feasible. Given the rarity of gynecologic PEComas, counseling reproductive-aged patients on fertility preservation remains challenging due to limited data on long-term outcomes.

Following the case presentation, with a novel fertility-sparing approach to a cervical perivascular epithelioid cell tumor, this report reviews the literature to summarize the current understanding of gynecologic PEComas. Key aspects reviewed will include diagnostic considerations, histopathologic evaluation, prognostic factors, and treatment options across all histopathologic classifications, with emphasis on fertility-sparing approaches.

## 2 Case Presentation

A reproductive-aged nulligravid woman with limited gynecologic care presented to a general gynecology office with abnormal uterine bleeding. The patient had no significant medical, surgical, or family history. Her first cervical cancer screening with a Pap smear was completed during workup for her presenting symptom of abnormal uterine bleeding. Upon presentation, she had had continuous vaginal spotting for four months. Her Pap smear demonstrated atypical glandular cells of undetermined significance. Physical exam was notable for a friable cervical mass extruding through the external cervical os. She underwent office-based endometrial and cervical biopsies. Pathology revealed cervical stroma with large epithelioid cells with eosinophilic cytoplasm and foamy mononuclear cells. Immunohistochemical staining was positive for Melan-A, HMB 45, and negative for CAM5.2, CD10, CD34, CD68, AE1/AE3, Desmin, p16, S100, ad SOX10. These findings were consistent with a PEComa.

The patient was referred to gynecology oncology for further surgical intervention. Preoperative evaluation included a computerized tomography (CT) scan of the chest, abdomen, and pelvis, which showed a 2 cm cervical mass, and pelvic magnetic resonance imaging (MRI) revealed a 12 × 17 mm enhancing mass in the endometrial canal. Previous pathology was sent to an outside lab for expert consultation, which confirmed a cervical PEComa through additional immunohistochemical stain. Additional staining was negative for smooth muscle actin, Calponin, Sox10, Caldesmon, and MS actin and positive for HMB45, Mart1/Melan A, and Desmin.

Hysteroscopic removal of the uterine mass and loop electrosurgical excision of the cervical mass were performed. Pathology confirmed a 3.1 cm cervical PEComa and an endometrial polyp. The PEComa was free from any malignant features. There was no necrosis, no mitoses in 50 high-power fields, and minimal cytologic atypia. There was no lymphovascular invasion or infiltrative growth pattern.

The patient successfully underwent fertility-sparing surgical excision of a benign cervical PEComa. At last follow-up she remained disease-free, with negative pelvic MRI scans at three, six, and twelve months. Given the rarity of cervical PEComas and the patient's strong desire for fertility preservation, her surveillance plan now involves close observation with twice-yearly pelvic MRI scans. This case highlights a novel use of a loop electrosurgical excision procedure to preserve fertility in those with gynecologic tumors, although ongoing surveillance is essential to ensure long-term safety.

## 3 Discussion

The diagnostic criteria for PEComas have evolved significantly over the past three decades. In 2002, the World Health Organization officially recognized PEComas as a distinct clinicopathologic entity. The foundation for this classification was laid in 1996 by Zamboni et al. [1], who identified histologic and immunohistochemical similarities between PEComas and other soft-tissue tumors. PEComas are characterized by epithelioid and spindle cells with a perivascular distribution, and immunoreactivity for melanocytic markers (HMB-45) and smooth muscle markers [3,[5], [6], [7]].

Early diagnosis remains difficult, as PEComas are often misdiagnosed as uterine fibroids or uterine sarcomas. Most gynecologic PEComas are diagnosed postoperatively following surgical resection and histologic examination [4]. In a case series by Shi et al. [8], only two of thirteen cases were diagnosed preoperatively. However, Tang et al. [8] described two cases of cervical PEComas diagnosed by conventional cervical smears. As of January 2023, only two cervical PEComa case reports had been reported in the English literature describing diagnosis based on conventional smears. Tang [9] described the first case of a cervical PEComa identified with liquid-based cytology. It involved a 55-year-old woman with an incidental finding of cervical PEComa on liquid-based cytology, confirmed on biopsy.

Tang's [9] case report highlighted an important diagnostic consideration: can cervical PEComas be reliably identified via liquid-based Pap smears? Liquid-based cytology offers a clear background, well-preserved cellular structure, and uniform distribution. In contrast, conventional smears present more diagnostic challenges. When cytologic features on ThinPrep raise suspicion for a PEComa, a definitive diagnosis requires biopsy, immunohistochemical staining, and possibly molecular testing. The potential for earlier diagnosis through liquid-based screening deserves further study, as it may improve outcomes for patients seeking fertility preservation.

Delayed or missed diagnoses can have profound consequences for fertility. For instance, Babayev et al. [10] described a uterine rupture secondary to an undiagnosed uterine PEComa, resulting in an abdominal pregnancy requiring a cesarean hysterectomy, permanently impacting future fertility. Such cases illustrate the high morbidity associated with undiagnosed and misdiagnosed PEComas, particularly in pregnancy. Nitahara et al. [11] reported the case of a 30-year-old who experienced an intraabdominal hemorrhage in the third trimester. Exploratory laparotomy revealed a ruptured PEComa of the round ligament. Fortunately, this patient delivered a healthy term infant without long-term fertility consequences; however, earlier diagnosis and surgical resection may have prevented a major open abdominal surgery. Other undiagnosed cases affecting pregnancy have been reported. Poomtavorn [12] reported a PEComa found at the time of cesarean section. Tilstra [13] reported a uterine PEComa misdiagnosed as a retained placenta.

Over the last three decades, risk stratification models for PEComas has been refined to predict outcomes based on histological factors. The importance of risk stratification is based on the varying risk of local recurrence and distant metastasis based on histopathologic features. In 2005, Folpe et al. [7] proposed a system which classified PEComas as benign, uncertain malignant potential, or malignant. A tumor was considered malignant if at least two of the following criteria are met: tumor size >5 cm, high nuclear grade, tumor necrosis, mitotic activity >1/50 HPF, and an infiltrative growth pattern with vascular invasion. Tumors lacking these features were considered benign, while tumors >5 cm with no additional adverse features were classified as having uncertain malignant potential. Bleeker et al. [14] further emphasized that tumor size >5 cm and mitotic rate >1/50 HPF are the strongest predictors of recurrence.

Currently, the World Health Organization defines malignant PEComas as exhibiting at least three of the following features: tumor size >5 cm, high mitotic rate (>1 mitosis per 50 high-power fields), high nuclear grade and cellularity, tumor necrosis, vascular invasion, and infiltrative growth pattern [7].

Schoolmeester et al. [15] examined sixteen cases of PEComa and found that all recurrent cases exhibited at least two malignant features. These findings reinforce the prognostic significance of histopathologic classification in guiding management. Understanding these histologic prognostic factors is crucial to balance surgical management with reproductive preservation.

Malignant PEComas are typically managed with total hysterectomy and bilateral salpingo-oophorectomy due to their metastatic potential. Levin [4] reviewed time to metastasis in patients with malignant PEComas and found that 40 % of patients with PEComas presented with metastatic disease at the time of diagnosis. The lungs are the most common site of metastasis [14]. On average, the time to identification of metastasis was as short as seventy-seven days postoperatively to as long as twenty years. In cases of ovarian malignant PEComas, recurrence occurred at twenty-five months [4].

Most cases of gynecologic PEComas in the literature, regardless of malignant potential, have been managed with total hysterectomy and bilateral salpingo-oophorectomy. However, for reproductive-aged patients with benign histopathology, fertility-sparing options have been explored in select cases. Next, a review of such case reports will be addressed with fertility-sparing approaches.

Bunch et al. [3] described a 19-year-old with malignant uterine PEComa who desired fertility preservation. She received mTOR inhibitor therapy following suboptimal surgical excision of the PEComa. After adjuvant therapy, she went under re-excision of the remaining tumor. Fifteen months after the operation she was free of metastatic disease.

Yamamoto et al. [2] reported a 24-year-old nulliparous woman with recurrent cervical PEComa. She underwent three fertility-preserving excisions but experienced multiple recurrences. Surgical excision technique was not reported.

Zemni et al. [16] discussed a patient with five years of infertility. The patient underwent an abdominal myomectomy of an intracavitary polypoid mass, initially diagnosed as a subserous myoma, but which was found to be a PEComa of uncertain malignant potential. The patient was monitored for six months without evidence of recurrence**.**

Shi et al. [8] performed a retrospective study of thirteen cases of PEComas with uncertain malignant potential. Eleven of the thirteen patients were treated surgically without chemotherapy or radiation. Eight of the patients underwent hysterectomy and five had fertility-sparing mass resections. There was no significant difference in disease-free survival between mass resection and hysterectomy groups, suggesting that fertility-sparing surgery may be a viable option in carefully selected cases.

A review by Gadducci [17] discussed the outcomes of a retrospective study performed by Liu et al. [18] One hundred and fourteen cases of PEComas of the female reproductive tract were evaluated. There were fifteen total cervical PEComas noted in the literature at that time. Since then, two additional case reports have been published. Of the seventeen published cervical PEComas, only two have been managed via excision. Disease recurrence was noted in both conservatively managed cases, with suspected metastasis in one. In contrast, seven of eight patients who underwent hysterectomy with bilateral salpingo-oophorectomy remained recurrence-free at thirty-six-month follow-up. Unfortunately, one patient had advanced disease with metastasis. This patient died nine months after total hysterectomy with bilateral salpingo-oophorectomy.

Fertility-sparing approaches for management of PEComas should remain the subject of discussion, due to limited data. While conservative surgical management has been attempted in select cases, recurrence rates appear to be higher compared to hysterectomy with bilateral salpingo-oophorectomy. Future studies should focus on optimizing patient selection, standardizing surveillance protocols utilizing MRI, and evaluating adjunctive therapies for improving outcomes in reproductive-aged patients with PEComas. Additional questions that remain pertinent include the accuracy of liquid-based cytology as a screening tool for early detection of cervical PEComas. Can fertility-sparing surgery be safely considered for both benign PEComas and those of uncertain malignant potential? What surveillance protocols are most appropriate for patients managed conservatively? Should MRI be included in the initial workup? How should reproductive-aged patients with benign PEComas, those of uncertain malignant potential, and malignant PEComas be counseled regarding fertility preservation options?

## 4 Conclusion

Fertility-sparing strategies should be at the forefront of management in women of reproductive age. More research is needed to establish evidence-based guidelines for fertility-sparing management. Until then, individualized patient counseling, risk stratification based on histopathologic features, and close surveillance remain critical components of care.

## Contributors

Laura Jore contributed to patient care, drafting the manuscript, undertaking the literature review and revising the article critically for important intellectual content.

Nneoma Edokobi contributed to drafting the manuscript and undertaking the literature review.

Ishani Deliwala contributed to drafting the manuscript and undertaking the literature review.

Marykate Potter contributed to drafting the manuscript and undertaking the literature review.

Meg Johnson contributed to drafting the manuscript and undertaking the literature review.

LucyBeth Nieves contributed to patient care and revising the article critically for important intellectual content.

All authors approved the final submitted manuscript.

## Patient consent

Written informed consent was obtained from the patient for publication of this case report.

## Provenance and peer review

This article was not commissioned and was peer reviewed.

## Funding

No funding from an external source supported the publication of this case report.

## Declaration of competing interest

The authors declare that they have no competing interest regarding the publication of this case report.
